# Supplementary figures and images for: Emergence of linkage between cooperative RNA replicators encoding replication and metabolic enzymes through experimental evolution
Source: PLoS Genet. 2023 Aug 4;19(8):e1010471. doi: 10.1371/journal.pgen.1010471 (PMC10431678; doi:10.1371/journal.pgen.1010471)

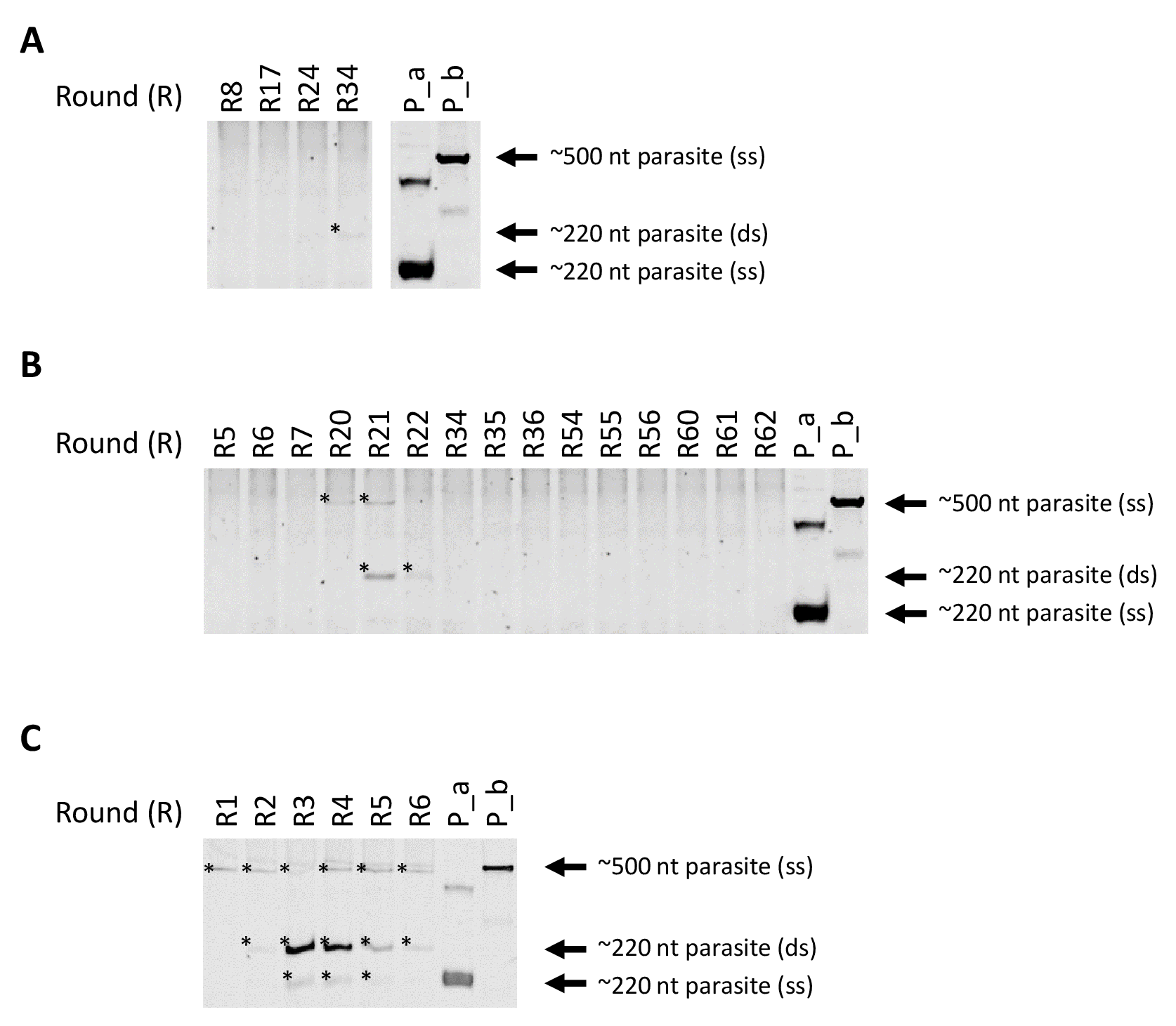

Supplement: S1 Fig — (A, B, C) Native PAGE of RNA mixtures during the long-term replication experiments shown in Figs 2A (A), 3A (B), and S5 (C). P_a and P_b are controls of commonly appearing parasitic RNAs of known sizes. Asterisks indicate the bands whose intensities were quantified. The expected parasitic RNA bands and their sizes are shown on the right. ss, single-strand. ds, double-strand. (TIF) [file pgen.1010471.s001.tif]

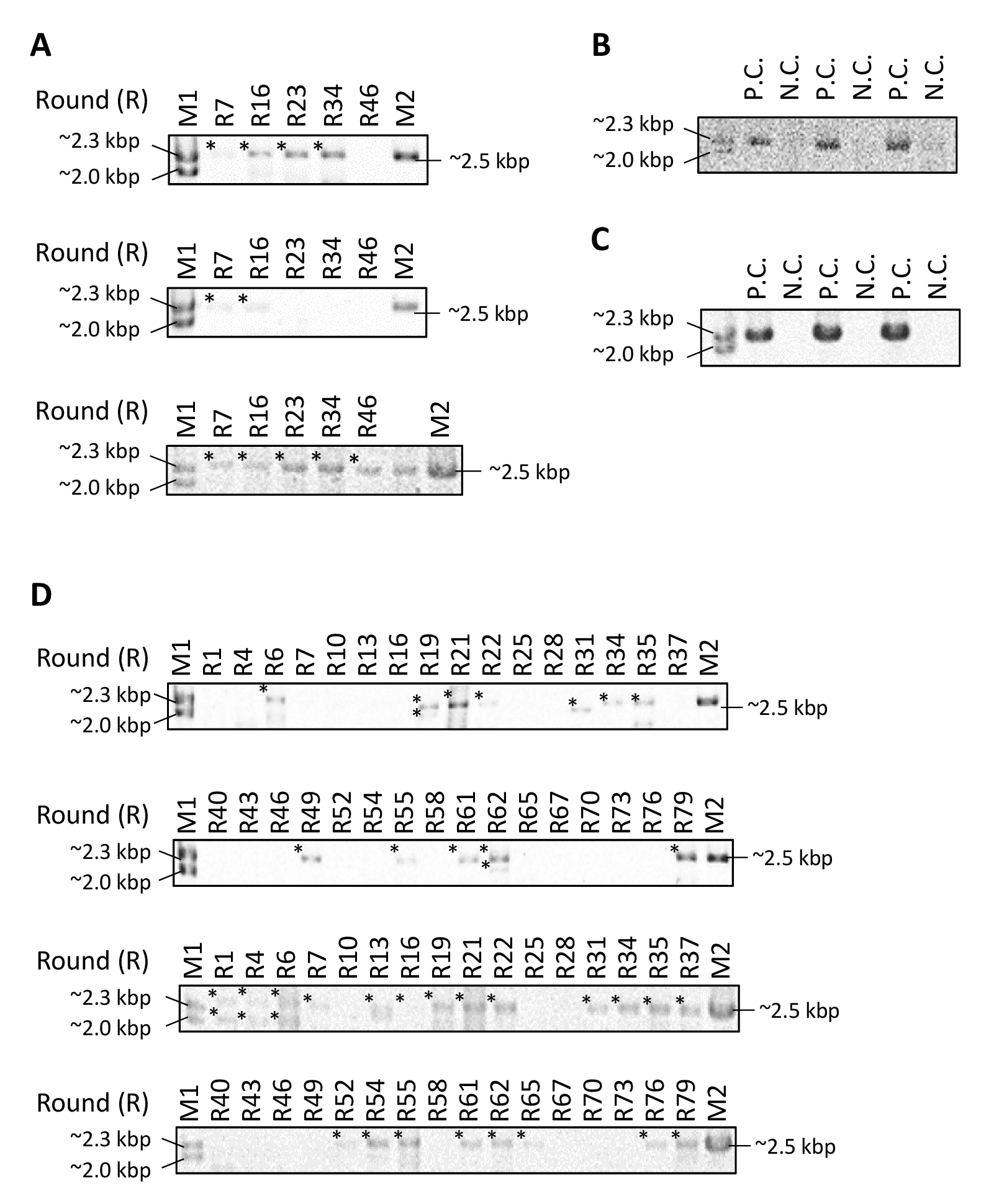

Supplement: S2 Fig — (A) Repeated RT-PCR using the primers that could detect 5′-rep-ndk-3′ for RNA samples in the first long-term replication experiments. The PCR products were analyzed by agarose gel electrophoresis. M1 and M2 are size markers. Asterisks indicate analyzed bands. (B) RT-PCR was performed three times in the same method for 0.1 nM RepNDK-RNA (P.C.) or a mixture of Rep- and NDK-RNAs (N.C., corresponding to 200 nM and 2000 nM in the long-term replication experiments, respectively). These RNAs were the representative clones obtained from the second long-term replication experiment. (C) RT-PCR was performed three times for the same P.C. and in the absence of RNA (N.C.). (D) Repeated RT-PCR for RNA samples in the second long-term replication experiments were performed and analyzed in the same method. (TIF) [file pgen.1010471.s002.tif]

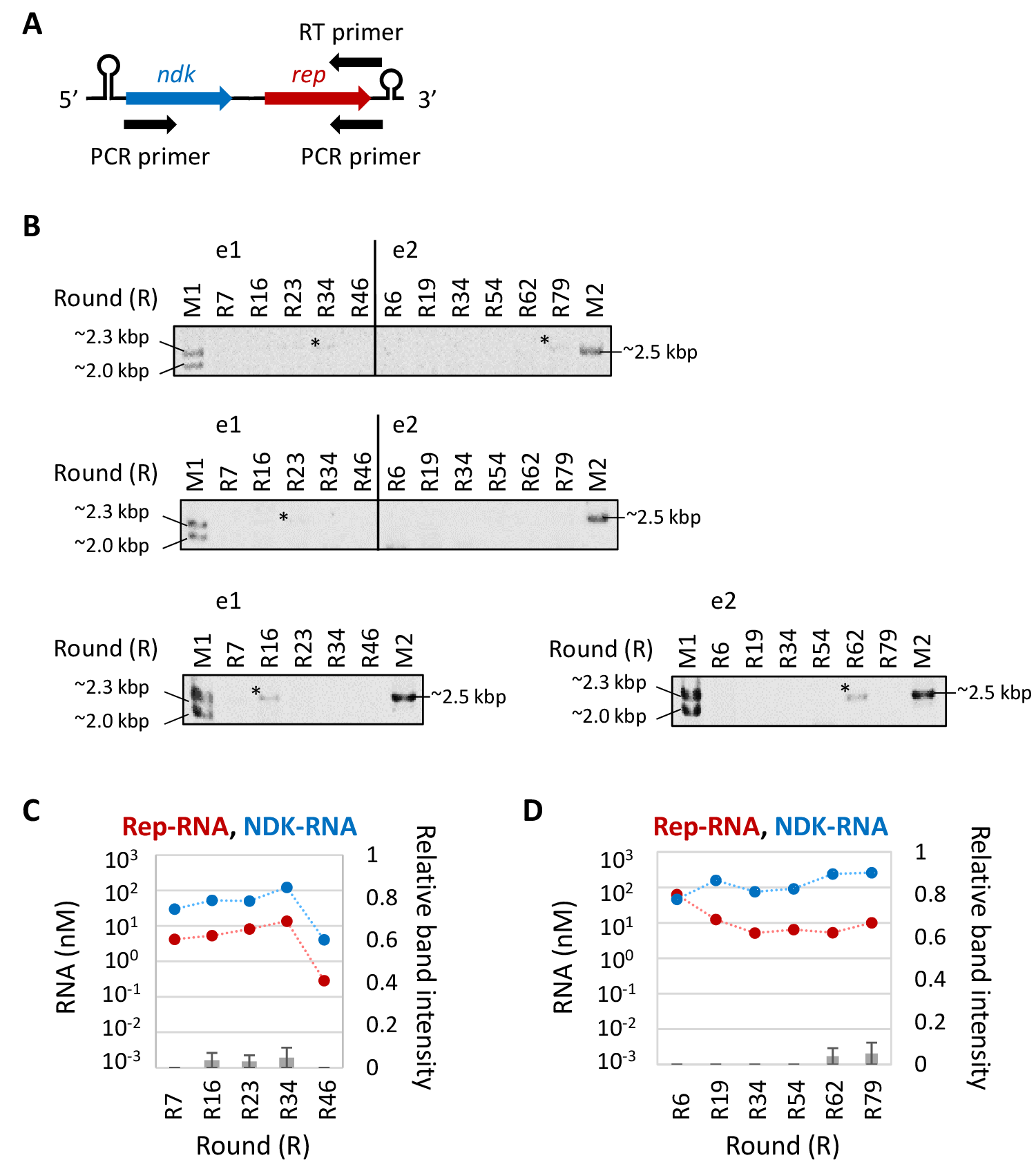

Supplement: S3 Fig — (A) RNA samples in the long-term replication experiments were subjected to RT-PCR using the primers that could detect 5′-ndk-rep-3′. (B) The PCR products were analyzed by agarose gel electrophoresis for RNA samples of the first (e1) and second (e2) long-term replication experiment (Figs 2A and 3A). All analyzed gels are shown (n = 3). M1 and M2 are size markers. Asterisks indicate analyzed bands. (C, D) Relative band intensities of the RT-PCR products, derived from the first (C) and second (D) long-term replication experiments, to M2 (gray bars, right axis), in comparison with Rep- and NDK-RNA concentrations (red and blue plots, left axis). Error bars indicate standard errors (n = 3). Dotted lines are plotted for visibility. (TIF) [file pgen.1010471.s003.tif]

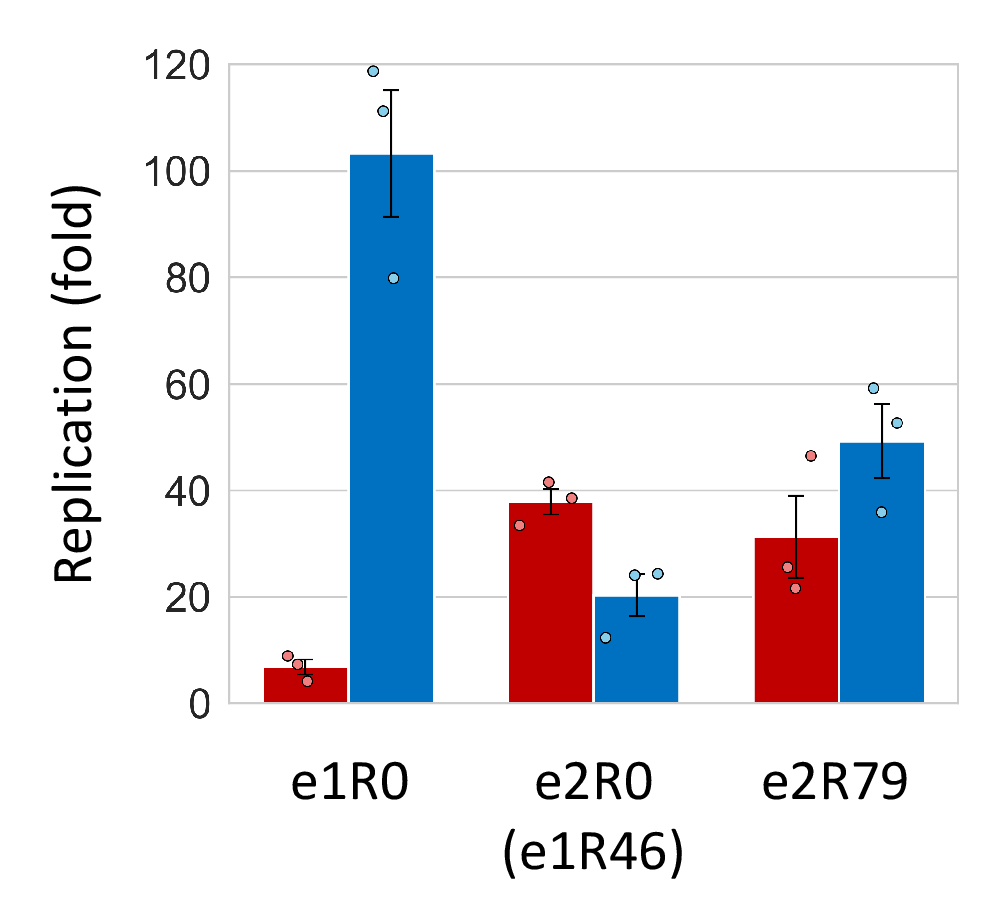

Supplement: S4 Fig — A pair of Rep- and NDK-RNAs (10 nM each) was incubated with a translation system in water-in-oil droplets at 37°C for 4 h, and their replication was measured by RT-qPCR. e1R0, e2R0 (e1R46), and e2R79 represent the ancestral RNA clones in the first long-term replication experiment (Fig 2A), the ancestral clones in the second long-term replication experiment (Fig 3A) (obtained at round 46 of the first experiment), and the clones obtained at round 79 of the second experiment, respectively. Error bars indicate standard errors (n = 3). (TIF) [file pgen.1010471.s004.tif]

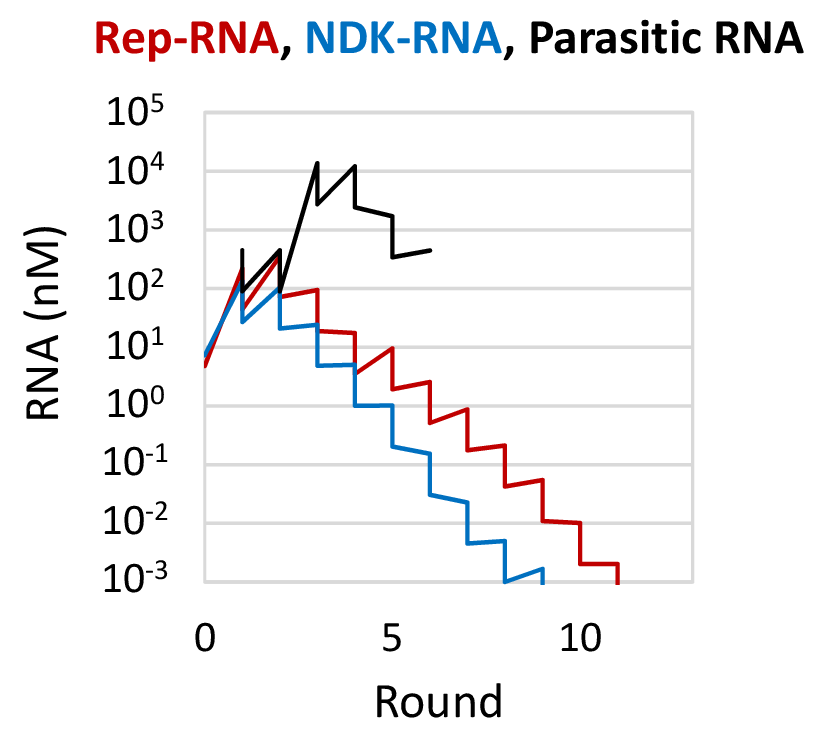

Supplement: S5 Fig — Changes in Rep-RNA (red), NDK-RNA (blue), and parasitic RNA (black) concentrations during the long-term replication experiment. The experiment was initiated with Rep- and NDK-RNAs “e2R0” and conducted without high temporal dilution. The replication step was performed at 37°C for 4 h. RNA concentrations were measured by RT-qPCR (Rep- and NDK-RNAs) or based on native PAGE (parasitic RNAs, S1C Fig). (TIF) [file pgen.1010471.s005.tif]

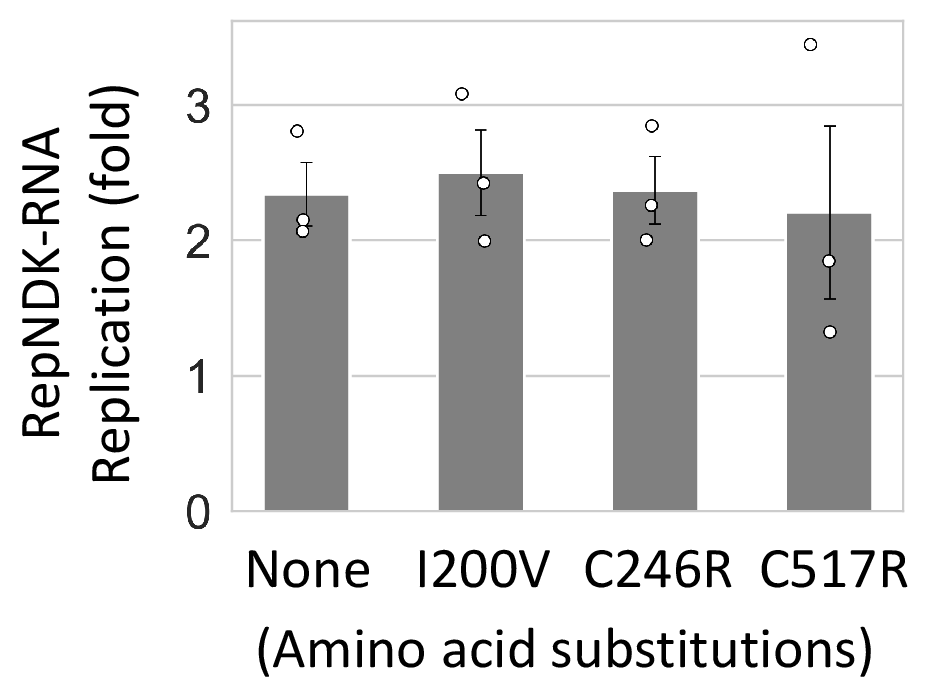

Supplement: S6 Fig — The experiments were performed in two steps. (1) A Rep-RNA clone (10 nM) with the truncated 3′ end was incubated at 37°C for 12 h in the absence of CTP to translate the replicase without RNA replication. Rep-RNA was constructed based on the ancestral Rep-RNA (“None”) or those containing one of the three dominant non-synonymous mutations that correspond to “I200V”, “C246R”, and “C517R” amino acid substitutions. (2) The translated replicase was mixed with the RepNDK-RNA clone (10 nM) and CTP to induce replication at 37°C for 4 h, while stopping further translation by the addition of streptomycin. The amount of RepNDK-RNA replication was measured by sequence-specific RT-qPCR. Error bars indicate standard errors (n = 3). (TIF) [file pgen.1010471.s006.tif]

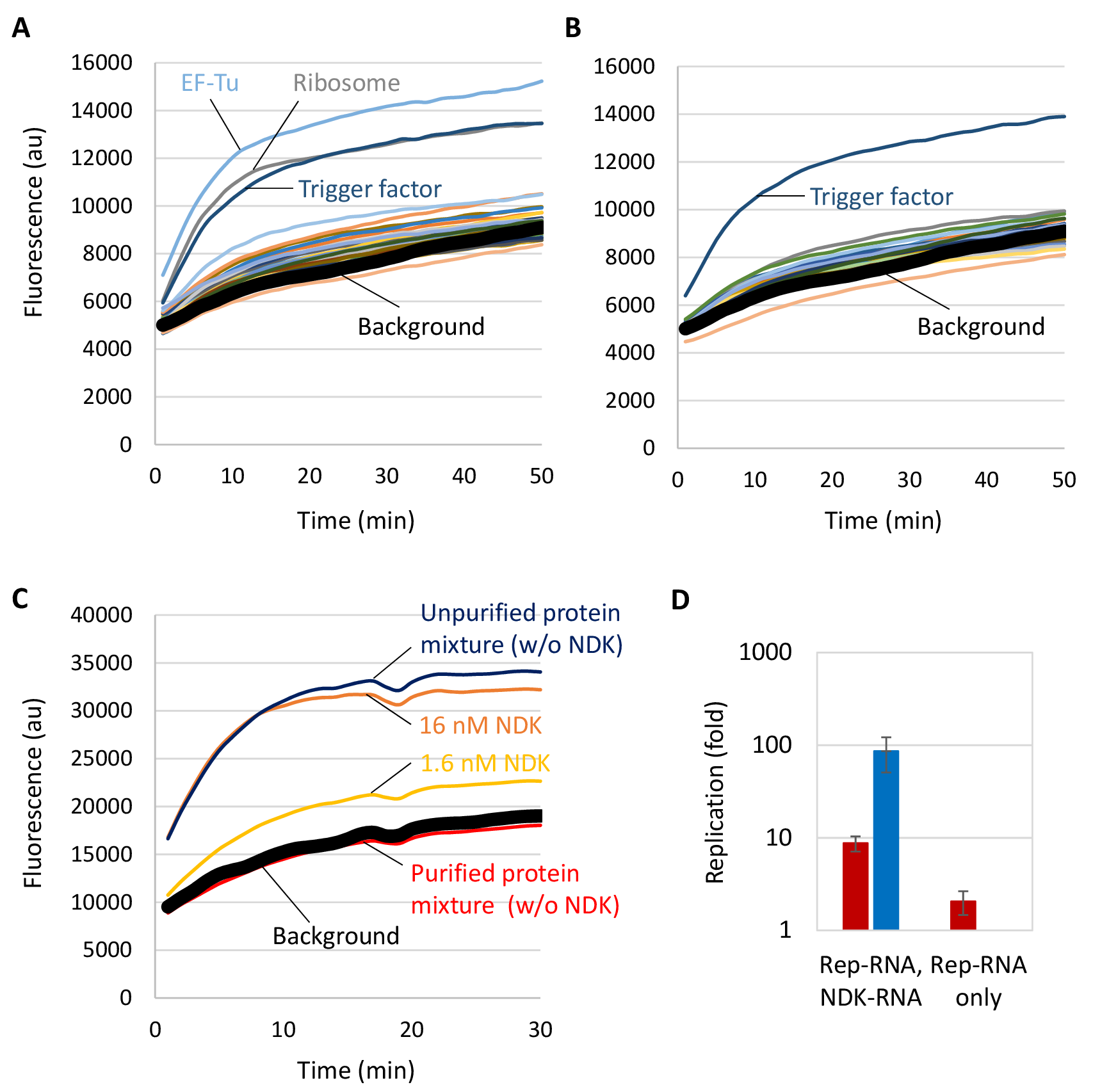

Supplement: S7 Fig — (A, B) The contamination level of NDK in each protein component before (A) and after (B) re-purification was measured as the increase in fluorescence using the ATP assay (see Materials and Methods). The protein names with high levels of contamination are indicated. The thick black lines indicate background fluorescence. (C) The contamination level of NDK in the mixture of all protein components before or after purification. The data were compared with the activity of 16 nM and 1.6 nM NDK. (D) 10 nM Rep-RNA (e1R0) was incubated in the presence or absence of 10 nM NDK-RNA (e1R0) with the purified translation system in water-in-oil droplets at 37°C for 4 h, and their replication was measured by RT-qPCR. Error bars indicate standard errors (n = 4). (TIF) [file pgen.1010471.s007.tif]

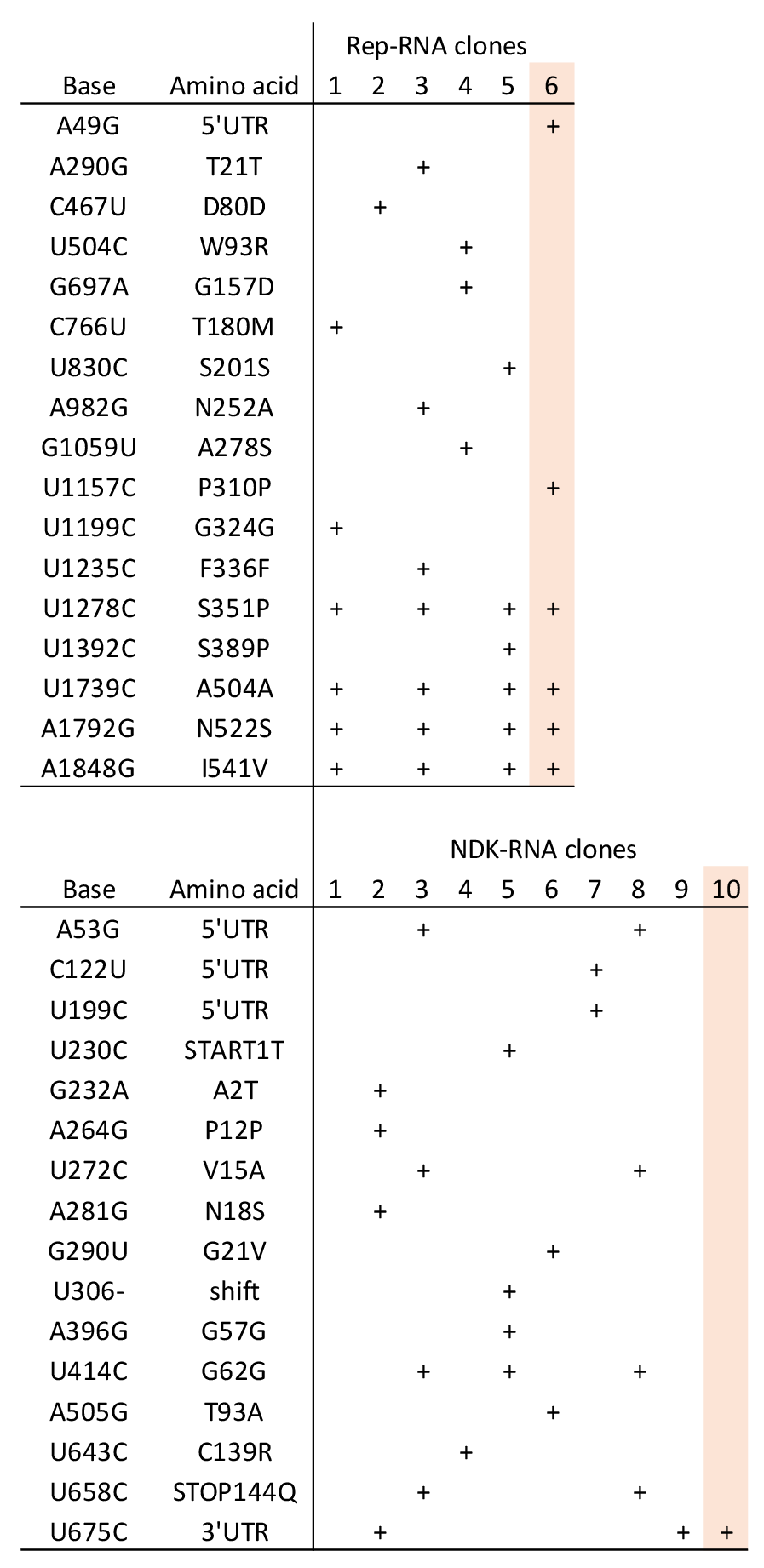

Supplement: S1 Table — The highlighted clones (Rep-RNA clone 6 and NDK-RNA clone 10) were used for further long-term replication experiments (Figs 3A and S5). (TIF) [file pgen.1010471.s008.tif]

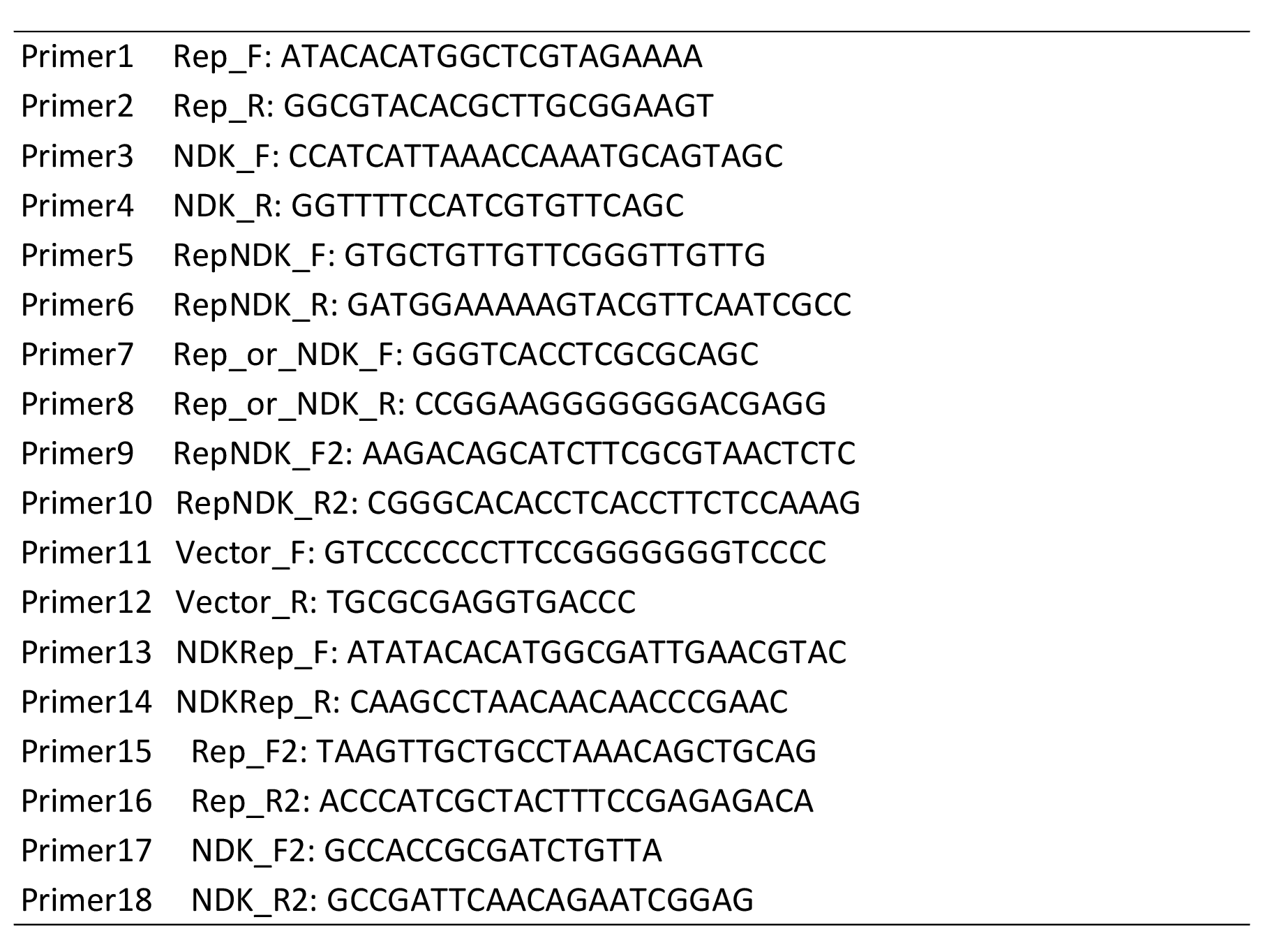

Supplement: S2 Table — (TIF) [file pgen.1010471.s009.tif]
