# Supplementary material for: Emergence of linkage between cooperative RNA replicators encoding replication and metabolic enzymes through experimental evolution
Source: PLoS Genet. 2023 Aug 4;19(8):e1010471. doi: 10.1371/journal.pgen.1010471 (PMC10431678; doi:10.1371/journal.pgen.1010471)
Supplement: Attachment — Submitted filename: 230629 point-by-point_PLOS Genet8.pdf [file pgen.1010471.s012.pdf]

Point-by-point response

Reviewer 1

This is a very stimulating paper. It provides confirmation of theory, but it also enriches the picture with valuable results. It deserves to be published. Some important comments for a minor revision:

Response:

We thank the reviewer for the positive evaluation of our manuscript. We have addressed all the comments raised by the reviewer.

Comment 1-1:

Emphasize that this is transient compartmentation. Fusion-fission cycles do not fit the model of a growing protocell population, as analyzed theoretically. This is important because the latter provides the most stringent selection for cooperation. The system used in this paper is transient, not only dynamically but also, presumably, in the early history of replicators. This is fully okay, but needs to be explained somewhat.

Response 1-1:

As pointed by the reviewer, fusion-fission cycles employed in our experiment was transient compartmentalization, different from growth-division cycles assumed in theoretical models. To describe this point, we added the following sentences in the Results section (subsection "Long-term replication experiments") where the fusion-fission cycles were mentioned for the first time.

"It should be noted that compartmentalization in this experimental procedure is transient because RNA molecules in different droplets can be mixed in every step of droplet fusion. More sustained compartmentalization could be achieved if compartments undergo growth instead of fusion, as implemented in previous theoretical studies [8–11]. Although these differences in compartmentalization may affect the evolutionary dynamics, both types of compartmentalization are conceivable in primitive compartments [20]."

Comment 1-2:

Chromosomes not only reduce the assortment load, but also automatically eliminate

competition between the LINKED replicators. And we do see that the two unlinked genes are competing. Furthermore, linkage presumably ensures a balanced production of translation products., which is likely to advantageous. Discuss!

Response 1-2:
We apologize for our insufficient description of the advantage of linkage. As the reviewer described, chromosome formation could also eliminate the competition between replicators and allow balanced production of translation products if the chromosome replicates alone. These advantages may have facilitated the evolution of linked (RepNDK-) RNA. We now discussed the two effects of chromosome formation as possible selective advantages of the linked RNA (now included in the Discussion). Because the former effect was well-known in previous theoretical studies, we also mentioned it briefly in the Introduction.

(Discussion, the second paragraph discussing the selection of linked RNA)
"In addition, unlike co-replicating Rep- and NDK-RNAs, independently replicable RepNDK-RNA has another advantage of avoiding the competition between RNA replicators and balancing the translation of the two encoded proteins."

(Introduction, the second paragraph describing previous theoretical studies)
"Another advantage is that chromosome formation eliminates the competition between unlinked replicators for the same resources."

Comment 1-3:
Going through proteins is different from the assumptions of the theory of being at an RNA world stage. In the latter case theory found that chromosomes with multigene families appear, but this is due to a direct dosage effect when RNAs act as enzymes -- not only as information carriers, as in the present paper.

Response 1-3:
As the reviewer pointed out, our RNA replication system contains proteins, and thus our study is not a direct confirmation of previous theoretical studies that assumed the RNA world. For example, the theoretical prediction based on dosage effect (Szilágyi et al., PLOS Genet., 2020) is not directly applicable to our work because the amount of translated proteins may be different from the copy number of each RNA. We also note that the current experimental methodology prohibited the detection of longer RNA with

more than two genes (e.g., Rep-Rep-NDK, Rep-NDK-Rep-NDK, etc.) as investigated in the theoretical study. We briefly mentioned these points in the Discussion (the third paragraph) as follows.

"It should be noted that some theoretical studies assumed the putative RNA world, where RNA (ribozyme) acts as both a catalyst and a carrier of genetic information [10, 11]. Thus, some theoretical predictions, such as the evolution of linked RNA to increase the copy number of ribozymes (i.e., dosage effect) [11] cannot be directly applied to our work in which RNA replicates using translated proteins."

Comment 1-4:

This leads me to the big unknown: the translation products. The expressed proteins evolve because their genes evolve, but here we see only the analysis of the latter. Granted one cannot do everything in a paper, but please tocuh upon this important point in the Discussion. I hope you will do this missing work sometime later. Note that the RNA for the replicase component can affect its own replicative phenotype as template as well as through the catalytic phenotype of its protein product.So far we see a compound effect.

Response 1-4:

As pointed out by the reviewer, the replication ability of an RNA can be divided into two factors: the ability of the RNA to be replicated as a template and the activity of the encoded replicase protein. In our study, we detected several dominant non-synonymous mutations (I200V, C246R, and C517R in Fig. 4A) in the replicase protein, although all dominant mutations detected in the NDK gene region were synonymous. Because the non-synonymous mutations may affect the function of the replicase, we newly investigated this possibility by replicating a RepNDK-RNA clone with replicases that contain each of the three mutations, as shown below (S6 Fig). The replicases were translated from Rep-RNA variants. The results showed that the effects of the amino-acid substitutions in the replicase were negligible for RepNDK-RNA replication. Therefore, the mutations may have contributed to the template ability.

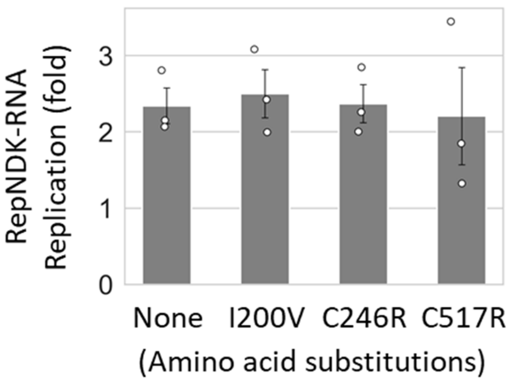

S6 Fig. Replicase activities on RepNDK-RNA replication analyzed by translation-uncoupled replication experiments. The experiments were performed in two steps. (1) A Rep-RNA clone (10 nM) with the truncated 3′ end was incubated at 37 °C for 12 h in the absence of CTP to translate the replicase without RNA replication. Rep-RNA was constructed based on the ancestral Rep-RNA ("None") or those containing one of the three dominant non-synonymous mutations that correspond to "I200V", "C246R", and "C517R" amino acid substitutions. (2) The translated replicase was mixed with the RepNDK-RNA clone (3 nM) and CTP to induce replication at 37 °C for 4 h, while stopping further translation by the addition of streptomycin. The amount of RepNDK-RNA replication was measured by sequence-specific RT-qPCR.

Relatedly, we changed the following paragraph in the Materials and Methods section. Changes were underlined.

In **"Plasmids and RNAs"**

"…<u>The plasmid encoding Rep-RNA clone e2R0 was further subjected to site-specific mutagenesis to introduce one of the three dominant amino acid substitutions (I200V, C246R, C517R).</u> For the RepNDK-RNA clone, the entire cDNA length was PCR-amplified from the corresponding plasmid. The cDNA of the RepNDK-RNA clone and the plasmids for all other RNA clones<u> except for ones with the amino-acid substitutions</u> were subjected to digestion with Sma I (Takara) and *in vitro* transcription with T7 RNA polymerase (Takara). <u>The cDNAs of Rep-RNA variants containing specific amino-acid substitutions were subjected to PCR amplification for truncation of 40 bp at the 3′ end before *in vitro* transcription.</u> All transcribed RNAs were purified using the RNeasy Mini Kit (QIAGEN)."

We also added the following paragraph in the Materials and Methods section.

New subsection: **Translation-uncoupled replication experiment**

"First, a truncated Rep-RNA (10 nM) containing a selected amino-acid substation was incubated at 37 °C for 12 h in the original translation system without CTP to induce the translation of the encoded replicase. Second, an aliquot of the reaction mixture was 5-fold diluted in the fresh mixture containing the translation system, 1.25 mM CTP, 30 µg/ml streptomycin, and 10 nM RepNDK-RNA clone, and subjected to further incubation. After 4 h incubation at 37 °C, the amount of RepNDK-RNA replication was measured by sequence-specific RT-qPCR as described above."

Comment 1-5:

Finally, it is appropriate to call attention to the fact that these replcators use a highly evolved, complex translation system provided by the experimenterm which can introduce unwanted side effects. But on the whole this is an important expperimental result.

Response 1-5:

We are grateful that the reviewer found our study important. As the reviewer noted, the presence of the translation system could introduce unwanted side effects. For example, the competition between Qβ replicase and the ribosome to use the same RNA strand in opposite direction may inhibit efficient RNA replication (Mizuuchi et al., ACS Synth. Biol., 2015)). This effect could become significant as the RNA becomes long by linkage because the frequency of collision between the Qβ replicase and the ribosome increases. To describe these points, we added the following sentences in the Discussion section (the third paragraph, and they immediately follow the discussion in Reponse 1-3).

"The translation process could also affect RNA replication in an unexpected way. For example, the competition between Qβ replicase and the ribosome to use the same RNA strand in opposite direction may inhibit efficient RNA replication [21], especially for long RNA molecules. Thus, the evolution of linkage may be more difficult than assumed in an RNA-only system."

Reviewer 2

In this article, Ueda et al. explore a very interesting idea concerning the emergence of primordial genomes. As the authors mention in the introduction, emergence of genomes would have conferred unique advantages and disadvantages to primordial replicators. They discuss these ideas briefly in the introduction and present the outline and objectives of the study in a clear and concise manner. The authors claim to demonstrate evolution of a 'linked' RNA genome, comprising of two (previously established) cooperative RNA replicators. The premise and relevance of the idea explored is relevant for audience interested in genetic and genomics research, particularly in the context of origin of life and evolution. However, I have multiple concerns and questions regarding the experimental methodology and data, as well as their description. Addressing these aspects is, in my opinion, necessary to improve the rigour of the manuscript, before it can be considered for acceptance in its current form.

Response:

We are grateful for the reviewer's time and effort in reading our manuscript and for raising the suggestion to improve the scientific rigor of the work. We are also happy that the reviewer found the idea we addressed very interesting. In the following parts, we have carefully addressed all the comments raised by the reviewer. We also note that all uncropped gel images and source data for the individual panels were included in the Source data file, as raised by the reviewer.

Comment 2-1:

The random emergence and disappearance of linked RNAs implies that all of the linked products do not bring about any fitness advantage (which seems to me the main selling point of the manuscript). This observation is also reflected in the lower replication rate and translation efficiency of the linked RNAs. As the authors suggest, the linked RNAs will likely be more competitive at higher RNA dilutions. The manuscript would greatly benefit if some simple competition experiment between linked and unlinked RNA species were performed at increasing levels of dilution.

Response 2-1:

We thank the reviewer for this important suggestion. According to the reviewer's suggestion, we newly performed competition experiments by combining Rep-, NDK-, and RepNDK-RNA clones. To imitate the condition of high RNA dilution (i.e., low RNA

concentrations), we varied initial RNA concentrations. We compared the replication of each RNA species when mixed at 1 nM each or 10 nM each, as shown below (Fig 5F). When mixed at 10 nM each, both Rep- and NDK-RNA clones replicated much more efficiently than the RepNDK-RNA clone, whereas at 1 nM each, the replication of the two unlinked RNAs decreased drastically compared to the linked (RepNDK-) RNA replication. In particular, the linked RepNDK-RNA clone replicated more than the unlinked Rep-RNA clone, suggesting that linked RNAs are more competitive at lower RNA concentrations (i.e., higher RNA dilutions in long-term replication experiments).

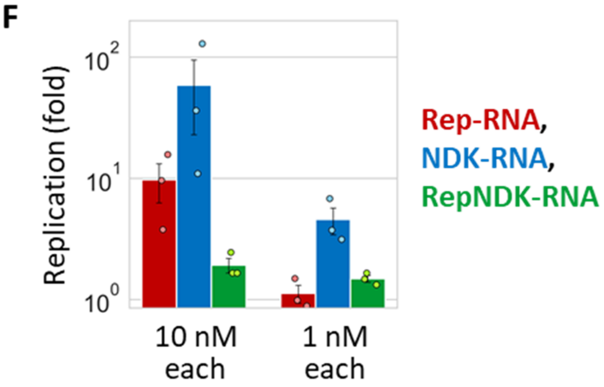

Fig 5. (F) The mixture of the Rep-, NDK-, and RepNDK-RNA clones (10 nM each or 1 nM each) was incubated with the translation system in water-in-oil droplets at 37 °C for 4 h, and their replication amounts were measured by RT-qPCR.

To describe these points, we added the following paragraph in the Results section (subsection "Biochemical characterization of RepNDK-RNA").

"Although the linked RepNDK-RNA clone replicated less efficiently than the pair of unlinked Rep- and NDK-RNAs, the linked RNA may have a relative selective advantage at low RNA concentrations because it can replicate on its own. We examined this possibility by incubating a set of Rep-, NDK-, and RepNDK-RNA clones mixed at low (1 nM each) or high (10 nM each) concentrations with a translation system in water-in-oil droplets at 37 °C for 4 h (Fig 5F). As expected, the replication of both Rep- and NDK-RNA clones decreased significantly at the 1 nM condition compared to the 10 nM condition (8.7- and 13-fold decreases, respectively). However, the RepNDK-RNA replication decreased only slightly (1.3-fold decrease) at the 1 nM RNA condition. Consequently, while the RepNDK-RNA clone replicated much less than the Rep- and NDK-RNA clones at the 10 nM condition, it replicated more than the Rep-RNA clone

under the 1 nM condition. These results confirmed that RepNDK-RNA can be more competitive at lower RNA concentrations."

Following these findings, we also changed sentences in the Discussion section, as follows (changes were underlined).

"How the linked RNA was maintained in the population while competing with the unlinked RNAs that replicate faster was not fully understood. One possible explanation could be that, unlike unlinked Rep- and NDK-RNAs, RepNDK-RNA can always replicate alone after redistribution in the droplet population, as suggested by theoretical studies [8,9,11]. This effect is expectedly enhanced when the stochastic mis-encapsulation of Rep- and NDK-RNAs becomes effective at low RNA concentrations [12] as supported by our experiment (Fig 5F)."

We also changed the corresponding Materials and Methods section ("Translation-coupled RNA replication experiments") as follows (changes were underlined).

"A pair of Rep- and NDK-RNA clones (10 or 1 nM each) and/or the RepNDK-RNA clone (10 or 1 nM) was incubated with the translation system in water-in-oil droplets at 37 °C for 4 h. … Primers 5 and 6, 15 and 16, and 17 and 18 were used for the RepNDK-RNA clone, the Rep-RNA clone, and the NDK-RNA clone, respectively (S2 Table).

Comment 2-2:
For the relative RT-PCR band intensities of the linked products in Figures 2C, 3C, S2C, and S2E, at least three replicates should be performed if the authors want to display bars. Otherwise, I fear that the statistics are insufficient for this type of semiquantitative analysis and can, at most, only be used for a binary yes/no detection. In light of the seemingly random nature of the emergence of the RT-PCR products, repeated negative controls might also be necessary to rule out sample cross-contaminations or RT-artefacts such as template switching.

Response 2-2:
We agree that our RT-PCR data were not quantitative because of the N = 1 experiments. We now additionally performed the experiments at least twice (at least N = 3 in total) for all concerned panels (Fig 2C, 3C, S2C, and S2E (S2 is now S3)), and combined all data as shown below(Fig 2B, 2C, 3B, 3C, new S2, and new S3). Negative control

experiments (RT-PCR with purified Rep- and NDK-RNAs (corresponding to 200 nM and 2000 nM, respectively, in the long-term replication experiment) or without any RNAs), performed 3 times, did not clearly show specific products (new S2B and S2C, shown below). All uncropped gel images were also included in the Source data file.

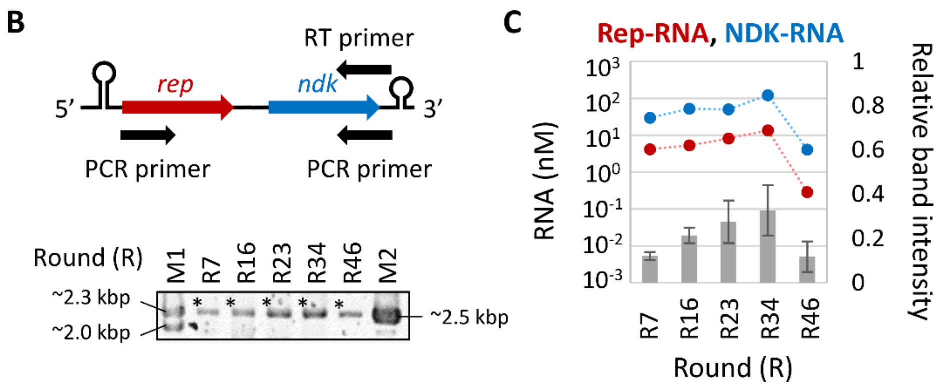

Fig 2. (**B**) RNA samples at the indicated rounds of the long-term replication experiment were subjected to RT-PCR, using primers that could detect 5′-*rep*-*ndk*-3′ (top), and PCR products were analyzed by agarose gel electrophoresis (bottom, a representative gel). M1 and M2 are size markers. Asterisks indicate analyzed bands. Repeated RT-PCR and negative control experiments were presented in S2 Fig. (**C**) Relative band intensities of the RT-PCR products to M2 (grey bars, right axis), in comparison with Rep- and NDK-RNA concentrations (red and blue plots, left axis). Error bars indicate standard errors (n=4). Dotted lines are plotted for visibility.

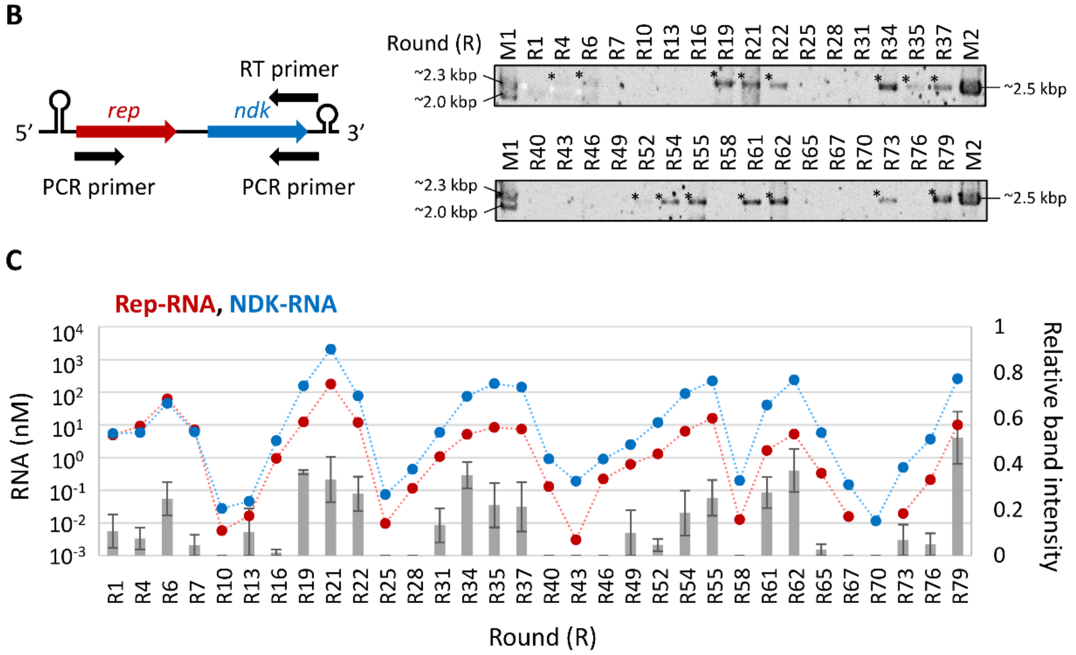

Fig. 3 (B) Detection of putative linked RNAs that harbor both *rep* and *ndk* genes (5′-*rep*-*ndk*-3′) in the RNA samples in every 1–3 rounds of the long-term replication experiment. RT-PCR was performed using the indicated primers (left), and PCR products were analyzed by agarose gel electrophoresis (right, representative gels). M1 and M2 are size markers. Asterisks indicate analyzed bands; white ones indicate shorter sizes. Repeated RT-PCR and negative control experiments were presented in S2 Fig. (C) Relative band intensities of RT-PCR products to M2 (grey bars, right axis), in comparison with Rep- and NDK-RNA concentrations (red and blue plots, left axis). Error bars indicate standard errors (n=3). Dotted lines are plotted for visibility.

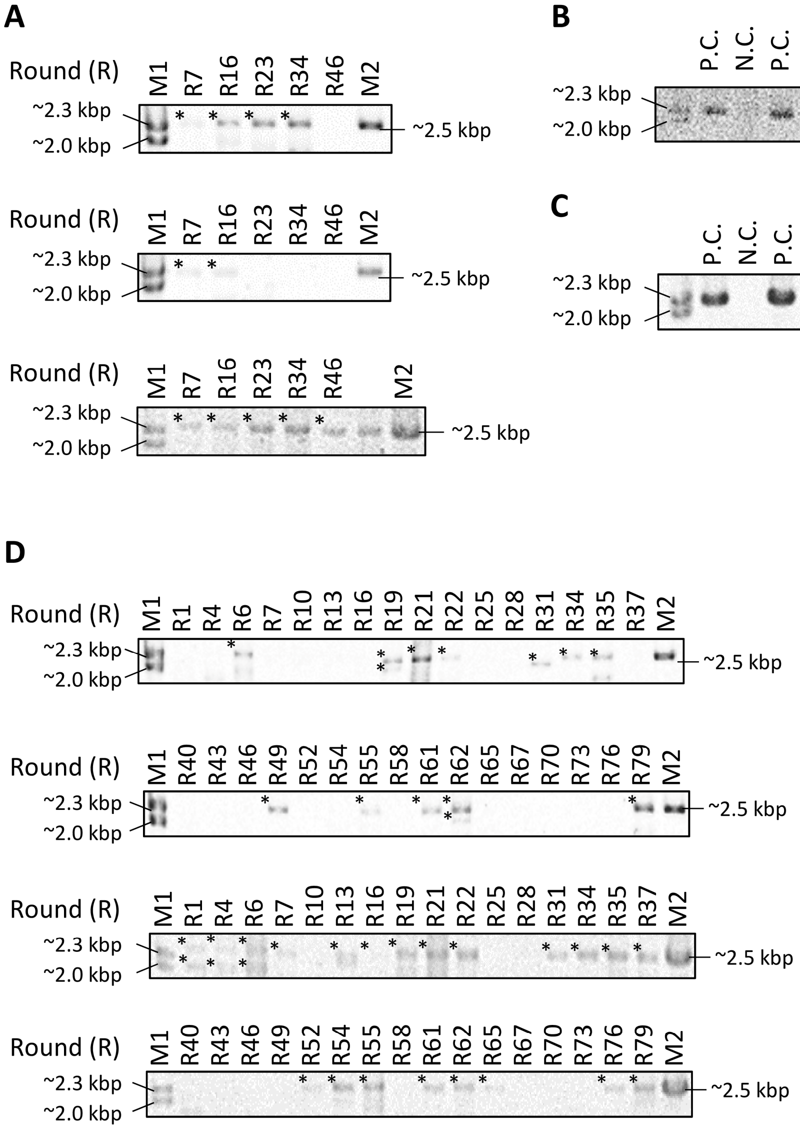

S2 Fig. Detection of putative linked RNAs harboring *rep* and *ndk* genes in this order. (A) Repeated RT-PCR using the primers that could detect 5′-*rep*-*ndk*-3′ for RNA samples in

the first long-term replication experiments. The PCR products were analyzed by agarose gel electrophoresis. M1 and M2 are size markers. Asterisks indicate analyzed bands. (B) RT-PCR was performed three times in the same method for 0.1 nM RepNDK-RNA (P.C.) or a mixture of Rep- and NDK-RNAs (N.C., corresponding to 200 nM and 2000 nM in the long-term replication experiments, respectively). These RNAs were the representative clones obtained from the second long-term replication experiment. (C) RT-PCR was performed three times for the same P.C. and in the absence of RNA (N.C.). (D) Repeated RT-PCR for RNA samples in the second long-term replication experiments were performed and analyzed in the same method.

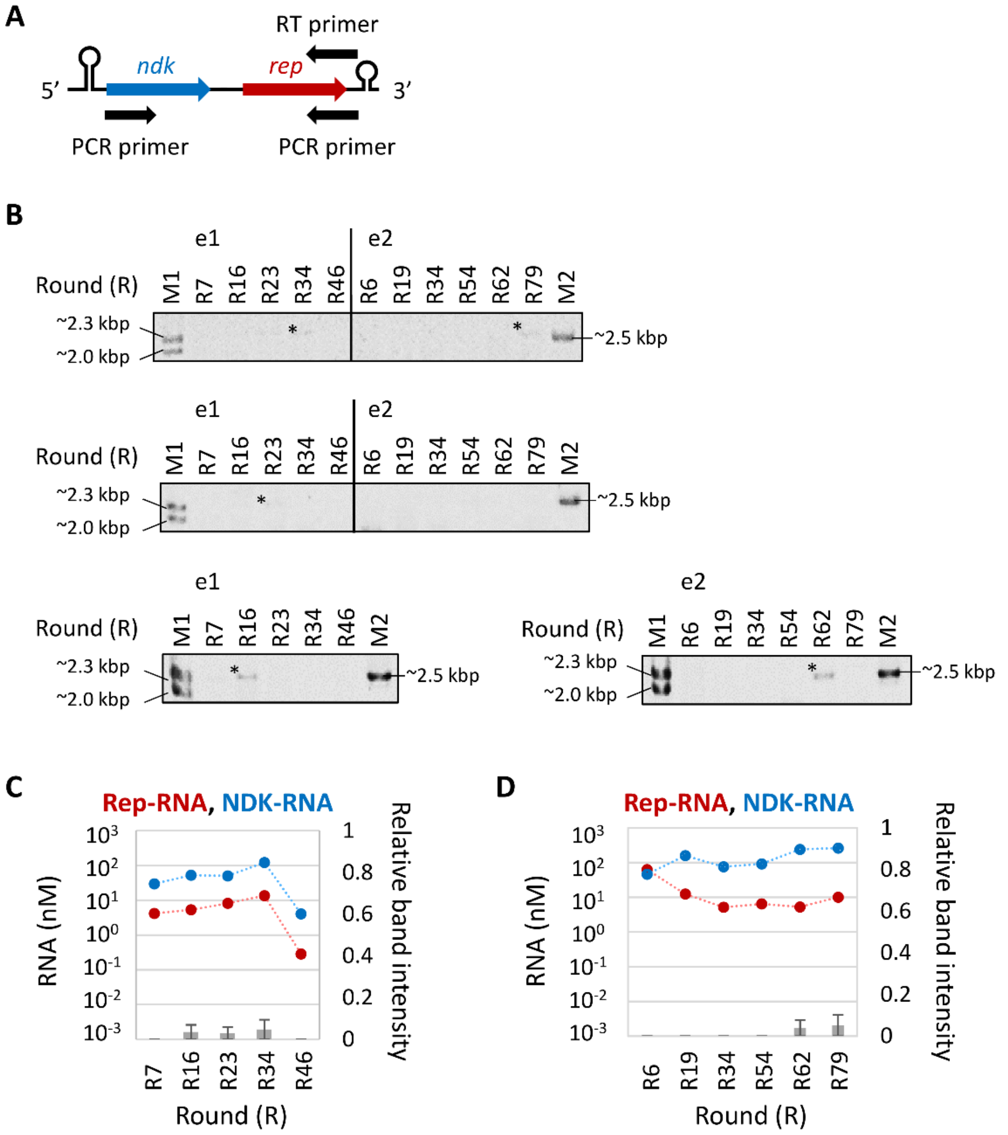

S3 Fig. Detection of putative linked RNAs harboring *rep* and *ndk* genes in the reverse order. (A) RNA samples in the long-term replication experiments were subjected to RT-

PCR using the primers that could detect 5′-*ndk*-*rep*-3′. (**B**) The PCR products were analyzed by agarose gel electrophoresis for RNA samples of the first (e1) and second (e2) long-term replication experiment (Fig 2A and 3A). All analyzed gels are shown (n=3). M1 and M2 are size markers. Asterisks indicate analyzed bands. (**C, D**) Relative band intensities of the RT-PCR products, derived from the first (C) and second (D) long-term replication experiments, to M2 (gray bars, right axis), in comparison with Rep- and NDK-RNA concentrations (red and blue plots, left axis). Error bars indicate standard errors (n=3). Dotted lines are plotted for visibility.

We also changed corresponding sentences in the Results section as follows (changes were underlined.)

(In "Long-term replication experiments," paragraph 3)
"Using agarose gel electrophoresis, we detected approximately 2.5 kbp products in all rounds (Fig 2B), with average band intensities roughly correlated with the base-10 logarithm of Rep- and NDK-RNA concentrations measured by RT-qPCR (correlation efficients are 0.77 and 0.81, respectively) (Fig 2C). We also performed RT-PCR using primers for RNA with the two genes linked in the reverse order (5′-*ndk*-*rep*-3′) (S3A Fig), but barely detected approximately 2.5 kbp products (S3B Fig, S3C Fig). These results suggested that longer RNAs that linked *rep* and *ndk* genes in a particular order appeared during the serial dilution cycle."

(In "Long-term replication experiments," paragraph 6)
"Next, we examined the appearance of a linked RNA by RT-PCR every 1–3 rounds, using primers that could detect 5′-*rep*-*ndk*-3′ (Fig 3B). We detected approximately 2.3–2.5 kbp PCR products at 15 of 32 analyzed rounds; the products appeared in all three trials of RT-PCR at rounds 6, 19, 21, 22, 34, 35, 55, 61, 62, and 79 (S2 Fig). We also detected seemingly smaller (2.0–2.3 kbps) products at a fraction of rounds, although their appearance was stochastic (S2 Fig). The relative intensities of the bands, if detected, were as strong as those in the first long-term replication experiment (Fig 2B) throughout the rounds. The average band intensities were roughly correlated with the base-10 logarithm of Rep- and NDK-RNA concentrations (correlation coefficients are 0.71 and 0.82, respectively) (Fig 3C). The highest band intensity was observed at the final round (79), at which Rep- and NDK-RNA concentrations were not the highest, suggesting that the linked RNA may have been selected in the final round. The concentration of a potentially linked RNA in the population at round 79 was estimated to be less than 1

nM, which is less than 10% of either of the unlinked RNAs, by RT-qPCR using primers that could detect the linkage region of 5′-*rep*-*ndk*-3′. We also performed RT-PCR at rounds 6, 19, 34, 54, 62, and 79 using primers for 5′-*ndk*-*rep*-3′ (S3B, S3D Fig), and barely detected approximately 2.5 kbps products.

Comment 2-3:

Given the huge population of sequences in each reaction, it seems questionable to me to draw phylogenetic conclusions about the origin/maintenance of the linked RNAs from the mutation patterns of only a handful of clones (6 to 16). In my opinion, the authors should either confirm their findings using high-throughput sequencing experiments (as they have already done in the past) or at least significantly increase the number of conventional clones they analyse.

Response 2-3:

We agree that the number of clones (16 for each of Rep-, NDK-, and RepNDK-RNAs) used for drawing phylogenetic trees were small (note that we obtained 16 NDK-RNA clones, although some of them were the same clones, and hence only 6 distinct clones). As per the reviewer's suggestion, we increased the number of clones to 47 for each of the three RNAs (total 141), sequenced them (Fig. 4A), and drew phylogenetic trees again (Fig 4B), as shown below. We analyzed all mutations that were detected in at least two clones. We found that the sequencing results were generally consistent with the previous result (based on 16 clones each), and suggested that some RepNDK-RNAs were forming partially separated lineages from the unlinked RNAs at least in the Rep-RNA region.

.

A

Rep-RNA region

Base	A825G	U963C	U1349C	A1604G	U1776C
Amino acid	I200V	C246R	V374V	Q459Q	C517R
Rep-RNA	38.3	91.5	55.3	57.4	36.2
RepNDK-RNA	89.4	95.7	6.4	6.4	89.4

Percentage: 100 – 0

NDK-RNA region

Base	A240G	U465C	G498A	U582C
Amino acid	E4E	V79V	L90L	G118G
NDK-RNA	34	38.3	34	68.1
RepNDK-RNA	10.6	21.3	10.6	31.9

Linkage region

Sites:	NDK region	
Base	G3A	T12G
RepNDK-RNA	83	87.2

B

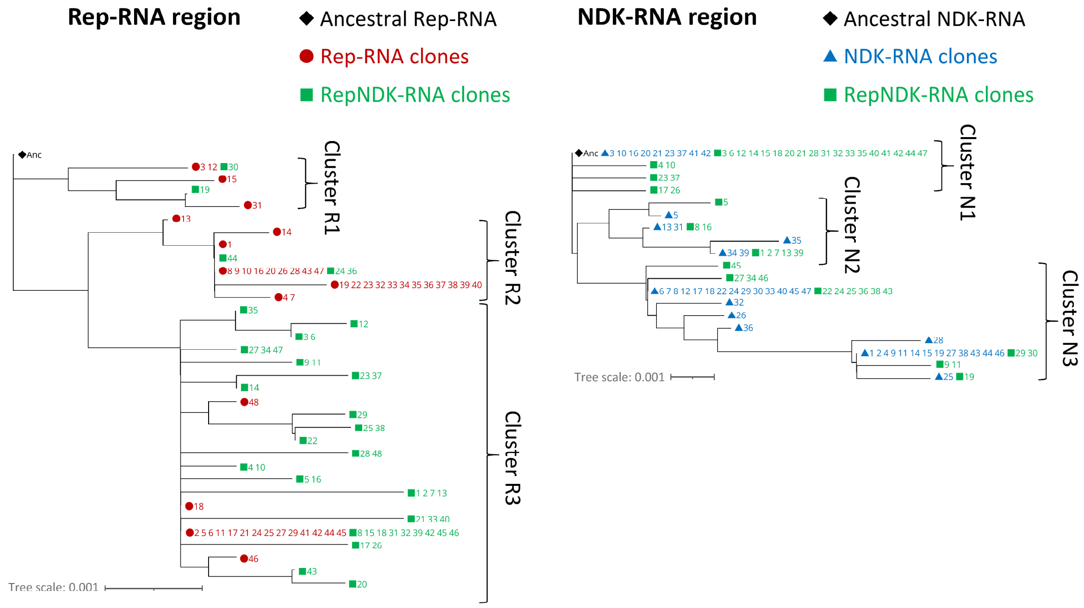

Fig 4. Accumulated mutations in the linked and unlinked RNAs. (A) Mutations detected in more than 30% of the 47 clones of any RNA species analyzed at round 79 are listed with their percentages. The sequence of the RepNDK-RNA clones was divided into Rep-RNA, NDK-RNA, and linkage regions for comparison with the Rep- and NDK-RNA clones. All detected mutations are listed in Supplementary Data. (B) Phylogenetic trees for each Rep- (left) and NDK- (right) RNA region were constructed from all the analyzed clones and mutations that were detected in at least two clones. Leaves representing different RNA species (Rep-, NDK-, or RepNDK-RNA) are marked with different colored symbols, followed by clone numbers. The ancestral Rep- and NDK-RNAs (Anc) were analyzed and displayed together.

To describe these points, we rewrote the corresponding places in the Result section as follows. Changes were underlined.

(In “Sequence analysis,” paragraph 1)

“In 45% of the RepNDK-RNA clones, Rep- and NDK-RNAs were simply connected end-to-end without deletion or insertion. In 26% of the clones, a single C was inserted between the connected Rep- and NDK-RNAs. At the NDK-RNA site around the linkage, we also detected G3A and U12G in 83% and 87% of the RepNDK-RNA clones, respectively (Fig 4A, linkage region).”

(In “Sequence analysis,” paragraph 2)

“The Rep- and NDK-RNA clones contained 3–9 and 0–7 mutations, respectively (Supplementary Data).”

(In “Sequence analysis,” paragraph 3)

“Fig 4A lists all mutations detected in more than 30% of the clones of any of the three RNA species (unlinked Rep- and NDK-RNAs and linked RepNDK-RNAs). The three types of RNAs accumulated different mutations at their corresponding sites. For example, A825G and U1776C were found in 38% and 36% of the Rep-RNA clones, respectively, whereas both mutations were detected in 89% of the RepNDK-RNA clones. In contrast, U1349C and A1604G were observed in 55% and 57% of the Rep-RNA clones, respectively, but detected in only 6% of the RepNDK-RNA clones. Similarly, A240G and G498A were detected in 34% of the NDK-RNA clones but in only 11% of the RepNDK-RNA clones. These different mutation patterns indicate that the majority of RepNDK-RNAs analyzed at round 79 were not derived from Rep- and NDK-RNAs of the same round. These results also suggested that RepNDK-RNA was maintained in the population for a certain period, long enough to form its own lineage.”

(In “Sequence analysis,” paragraph 4)

“We further illustrated the evolutionary relationships between Rep- or NDK-RNA and RepNDK-RNA using phylogenetic trees for each of the Rep- and NDK-RNA regions (Fig 4B). The three RNA species are represented by different symbols. For the Rep-RNA region (Fig 4B, left), there were three clusters (Clusters R1–3). Cluster R1 consisted of four unlinked Rep-RNA clones (red circles) and two linked RepNDK-RNA clones (green squares). Cluster R2 mainly contained unlinked Rep-RNA clones; it consisted of 26 Rep-RNA clones and only three RepNDK-RNA clones. In contrast, Cluster R3 mainly contained linked RepNDK-RNA clones; it consisted of 17 Rep-RNA clones and 42 RepNDK-RNA clones. Furthermore, the unlinked Rep-RNA clones were located near the

root of the branches in Cluster R3, compared to the linked RepNDK-RNA clones. These results suggested that RepNDK-RNA was forming distinct lineages from the unlinked RNAs in Cluster R3. For the NDK-RNA region (Fig 4B right), there were also three clusters, However, unlike the Rep-RNA region, the unlinked NDK-RNA and linked RepNDK-RNA clones were mixed well, indicating that the characteristic mutations of linked RNA were not present in the NDK-RNA region."

(In "Biochemical characterization of RepNDK-RNA," paragraph 1)
"we selected a clone containing only the most common mutation set (A825G, T963C, U1776C, and -2042C in the Rep-RNA region and G3A and U12G in the NDK-RNA region), which was found in 26% of the 47 clones. For comparison, we also chose a pair of unlinked Rep- and NDK-RNAs containing only the most common mutation sets at round 79. The selected Rep-RNA contained U963C, U1349C, and A1604G, found in 55% of the Rep-RNA clones, whereas the selected NDK-RNA contained A240G, U465C, G498A, and U582C, found in 32% of the NDK-RNA clones."

Comment 2-4:
More quantitative details on the RNA concentrations is sorely needed throughout the manuscript. E.g. In lines 144, 172 and 175., the amount of RNA is described in very vague terms only, which affects the scientific rigour of the manuscript.

Response 2-4:
We apologize for our insufficient description of RNA concentrations. The concerned parts described relationships between band intensities and RNA concentrations. To provide qquantitative information on the relationships, we calculated correlation coefficients between the two factors (more precisely, band intensities and the base-10 logarithm of RNA concentrations). These changes were detailed in Response 2-2. We also added more quantitative descriptions throughout the manuscript.

Comment 2-5:
What are the effects of the I200V, C246R, and C517R amino acid substitutions on the activity of Rep? Are they associated with a fitness advantage with respect to the replication of the linked genomes?

Response 2-5:
It is possible that the amino acid substations affect the activity of the replicase, possibly

associated with the fitness of the linked RNA. Therefore, we decided to investigate how a RepNDK-RNA clone was replicated by replicase variants containing each of the three amino acid substations. To this end, we performed translation-uncoupled experiments in the following two steps. First, modified Rep-RNA variants containing only each of the three corresponding non-synonymous mutations were translated without being replicated (the replicase recognition site at the 3′ end was truncated, and CTP was omitted). Second, the reaction mixture was mixed with the RepNDK-RNA and CTP to initiate replication. As shown below (S6 Fig), RepNDK-RNA was replicated nearly equally by the original and three mutant replicases, indicating that any of the single amino acid substations did not contribute to the replicase activity. The corresponding non-synonymous mutations may have been attributed to the efficiency of RepNDK-RNA to be a template for replication.

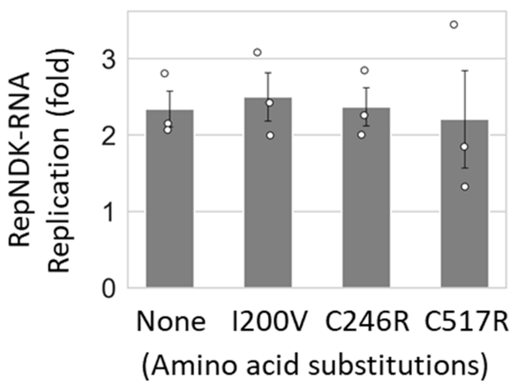

S6 Fig. Replicase activities on RepNDK-RNA replication analyzed by translation-uncoupled replication experiments. The experiments were performed in two steps. (1) A Rep-RNA clone (10 nM) with the truncated 3′ end was incubated at 37 °C for 12 h in the absence of CTP to translate the replicase without RNA replication. Rep-RNA was constructed based on the ancestral Rep-RNA ("None") or those containing one of the three dominant non-synonymous mutations that correspond to "I200V", "C246R", and "C517R" amino acid substitutions. (2) The translated replicase was mixed with the RepNDK-RNA clone (10 nM) and CTP to induce replication at 37 °C for 4 h, while stopping further translation by the addition of streptomycin. The amount of RepNDK-RNA replication was measured by sequence-specific RT-qPCR. Error bars indicate standard errors (n=3).

To describe these points, we added the following paragraph in the Results section (subsection "Biochemical characterization of RepNDK-RNA").

"We also investigated the effect of the three dominant amino acid substitutions (I200V, C246R, C517R) in the replicase (Fig. 4A) on RepNDK-RNA replication. We performed translation-uncoupled experiments in the following two steps. First, a modified Rep-RNA variant that contains each of the three non-synonymous mutations corresponding to the amino acid substitutions was incubated to induce translation of the replicase. In this reaction, the replication of the Rep-RNA was precluded because we removed the 3′ end (replicase recognition site) of the Rep-RNA and omitted CTP in the reaction mixture Second, we mixed the reaction mixture with the RepNDK-RNA and CTP to initiate RepNDK-RNA replication. We observed only negligible effects of any single amino acid substitutions on the ability of the replicase for RepNDK-RNA replication (S6 Fig). Thus, the non-synonymous mutations may have contributed to different factors such as the ability of RepNDK-RNA to be a template for replication."

We also changed the following paragraph in the Materials and Methods section. Changes were underlined.

In **"Plasmids and RNAs"**
"…The plasmid encoding Rep-RNA clone e2R0 was further subjected to site-specific mutagenesis to introduce one of the three dominant amino acid substitutions (I200V, C246R, C517R). For the RepNDK-RNA clone, the entire cDNA length was PCR-amplified from the corresponding plasmid. The cDNA of the RepNDK-RNA clone and the plasmids for all other RNA clones except for ones with the amino-acid substitutions and a control RNA without substitutions were subjected to digestion with Sma I (Takara) and *in vitro* transcription with T7 RNA polymerase (Takara). The cDNAs of Rep-RNA variants containing specific amino-acid substitutions and the control Rep-RNA were subjected to PCR amplification for truncation of 40 bp at the 3′ end before *in vitro* transcription. All transcribed RNAs were purified using the RNeasy Mini Kit (QIAGEN)."

Finally, we added the following paragraph in the Materials and Methods section.

New subsection: **Translation-uncoupled replication experiment**
"First, a truncated Rep-RNA (10 nM) containing a selected amino-acid substation was incubated at 37 °C for 12 h in the original translation system without CTP to induce the translation of the encoded replicase. Second, an aliquot of the reaction mixture was 5-fold diluted in the fresh mixture containing the translation system, 1.25 mM CTP, 30

µg/ml streptomycin, and 10 nM RepNDK-RNA clone, and subjected to further incubation. After 4 h incubation at 37 °C, the amount of RepNDK-RNA replication was measured by sequence-specific RT-qPCR as described above."

Comment 2-6:

Apart from these, I have some minor comments:

- Figure S5. To me it seems unclear to which extend the arbitrary fluorescence increase is associated with residual NDK activity as there are no reference points (baseline / positive control). I.e. is the residual activity observed after re-purification still significant in particular if all proteins are combined again? Is there still detectable replication of Rep-RNA in this background without NDK-RNA?

Response 2-6:

We apologize for our unclear data visualization. The data presented in Figure S5 (now S7) were after subtraction of the baseline. We now included raw data with a baseline for all panels (S7, shown below). We also performed the same experiment with a positive control (purified NDK) and mixtures of all protein components before and after purification, shown below (S7C). Fig. S7C shows that the residual NDK activity in the purified protein mixtures was at background level and much lower than the original protein mixtures (without NDK). The comparison with a dilution series of purified NDK demonstrated that the residual activity was lower than that of 1.6 nM NDK. Using the purified proteins, we also investigated the background replication of Rep-RNA (Fig. S7D). The Rep-RNA replication was barely detectable in the absence of NDK-RNA, confirming our purification step successfully removed most of the residual NDK activity.

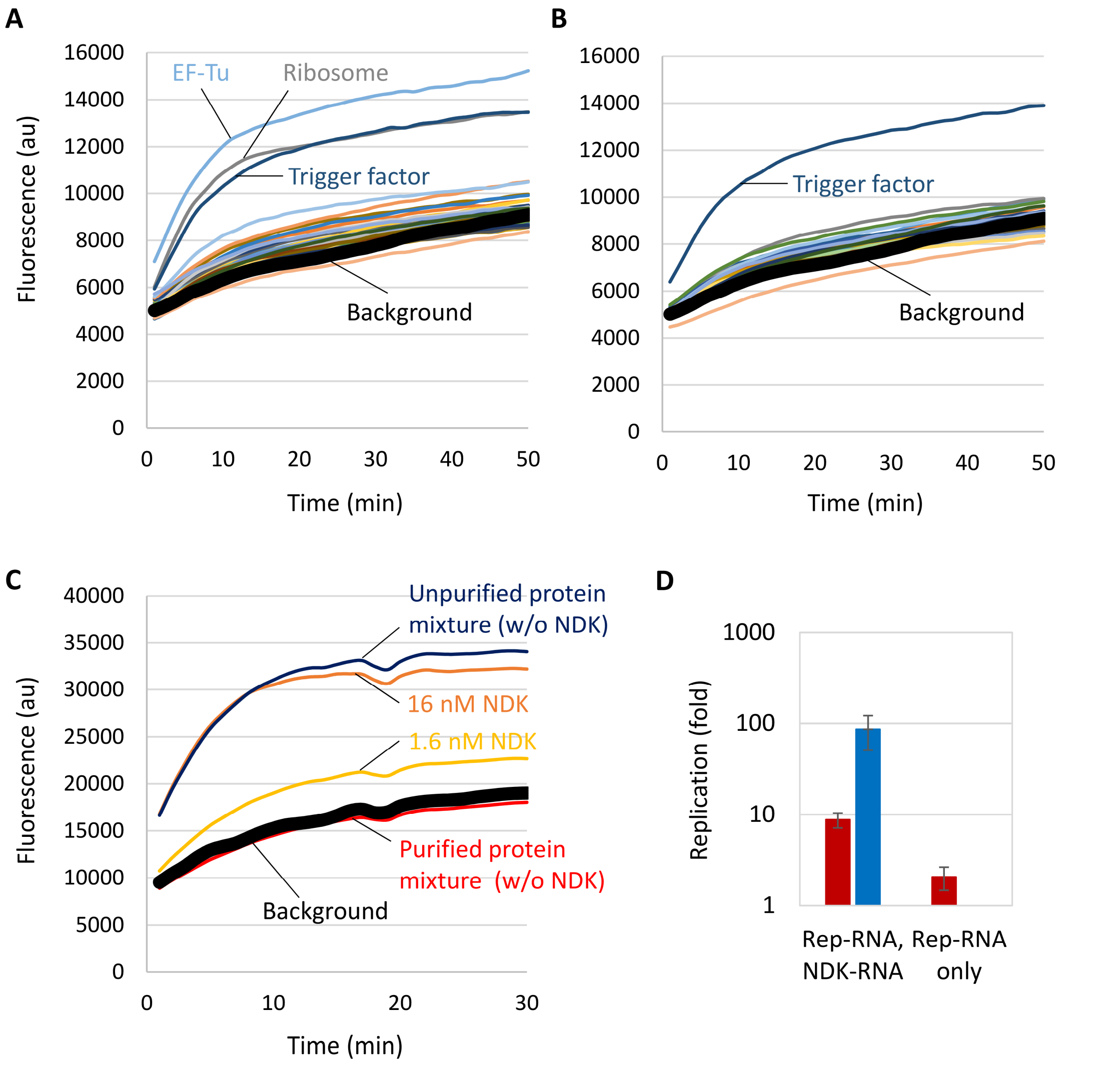

S7 Fig. Contamination levels of NDK in the translation system. (A, B) The contamination level of NDK in each protein component before (A) and after (B) re-purification was measured as the increase in fluorescence using the ATP assay (see Materials and Methods). The protein names with high levels of contamination are indicated. The thick black lines indicate background fluorescence. (C) The contamination level of NDK in the mixture of all protein components before or after purification. The data were compared with the activity of 16 nM and 1.6 nM NDK. (D) 10 nM Rep-RNA (e1R0) was incubated in the presence or absence of 10 nM NDK-RNA (e1R0) with the purified translation system in water-in-oil droplets at 37°C for 4 h, and their replication was measured by RT-qPCR. Error bars indicate standard errors (n=4).

Comment 2-7:

- sentence in line 295-297. Isn't this counter to experimental findings, considering the

emphasis authors place on the dilution step? Please clarify.

Response 2-7:

We agree that the concerned sentence was ambiguous. We meant that we do need the control of dilution (when the concentrations of cooperative RNAs become high), but we do not need as strict control of dilutions as employed in the previous study (Mizuuchi & Ichihashi, Nat. Ecol., Evol., 2018). In the previous study, we controlled the dilution rate every round to keep the concentration of the two cooperative RNAs below a certain level (1 nM). However, in this study, we found that high temporal dilution is sufficient because the propagation of parasitic RNAs in the droplet population was not as fast as previously thought, and the concentration of the cooperative RNAs can be higher than 100 nM, at least tentatively. To describe these points, we rewrote the paragraph as follows (changed parts are underlined).

"We also developed a new method that allows the long-term replication of cooperative RNAs, even if the RNA concentrations increase tentatively and parasitic RNA emerges. Our previous study suggested that maintaining a relatively low RNA concentration was crucial for sustained replication of the cooperative system, and continuous RNA replication was achieved only by controlling the dilution rate at every replication round and maintaining the two cooperative RNAs below 1 nM [12]. However, the present study demonstrated that sustainable cooperative RNA replication does not require as strict regulation of RNA concentration as previously employed, and the cooperative RNAs could be more than 100 nM, at least occasionally. Thus, although tentative high dilution was still necessary, the molecular cooperation was more robust than previously thought. A higher RNA concentration also increases the chance of recombination or ligation of RNAs and may facilitate the evolution of a primitive chromosome."
